# Outdoor artificial light at night, air pollution, and risk of childhood acute lymphoblastic leukemia in the California Linkage Study of Early-Onset Cancers

**DOI:** 10.1038/s41598-022-23682-z

**Published:** 2023-01-11

**Authors:** Charlie Zhong, Rong Wang, Libby M. Morimoto, Travis Longcore, Meredith Franklin, Tormod Rogne, Catherine Metayer, Joseph L. Wiemels, Xiaomei Ma

**Affiliations:** 1 Department of Population and Public Health Sciences, Keck School of Medicine, University of Southern California, Los Angeles, CA USA; 2 Department of Chronic Disease Epidemiology, Yale School of Public Health, 60 College St, New Haven, CT 06520 USA; 3 School of Public Health, University of California, Berkeley, Berkley, CA USA; 4 Institute of the Environment and Sustainability, University of California, Los Angeles, Los Angeles, CA USA; 5 Department of Statistical Sciences, University of Toronto, Toronto, ON Canada; 6 Gemini Center for Sepsis Research, Department of Circulation and Medical Imaging, NTNU, Norwegian University of Science and Technology, Trondheim, Norway; 7 Centre for Fertility and Health, Norwegian Institute of Public Health, Oslo, Norway

**Keywords:** Cancer epidemiology, Acute lymphocytic leukaemia, Environmental impact

## Abstract

Acute lymphoblastic leukemia (ALL) is the most common type of cancer in children (age 0–14 years); however, the etiology remains incompletely understood. Several environmental exposures have been linked to risk of childhood ALL, including air pollution. Closely related to air pollution and human development is artificial light at night (ALAN), which is believed to disrupt circadian rhythm and impact health. We sought to evaluate outdoor ALAN and air pollution on risk of childhood ALL. The California Linkage Study of Early-Onset Cancers is a large population-based case–control in California that identifies and links cancer diagnoses from the California Cancer Registry to birth records. For each case, 50 controls with the same year of birth were obtained from birth records. A total of 2,782 ALL cases and 139,100 controls were identified during 2000–2015. ALAN was assessed with the New World Atlas of Artificial Night Sky Brightness and air pollution with an ensemble-based air pollution model of particulate matter smaller than 2.5 microns (PM_2.5_). After adjusting for known and suspected risk factors, the highest tertile of ALAN was associated with an increased risk of ALL in Hispanic children (odds ratio [OR] = 1.15, 95% confidence interval [CI] 1.01–1.32). There also appeared to be a borderline association between PM_2.5_ level and risk of ALL among non-Hispanic White children (OR per 10 µg/m^3^ = 1.24, 95% CI 0.98–1.56). We observed elevated risk of ALL in Hispanic children residing in areas of greater ALAN. Further work is needed to understand the role of ALAN and air pollution in the etiology of childhood ALL in different racial/ethnic groups.

Leukemia is the most common type of cancer in children (age 0–14 years), accounting for approximately one third of all cancers in this age group^1^. The most common subtype (80%) of childhood leukemia is acute lymphoblastic leukemia (ALL), which is a disease of the immune system caused in part by mutations that occur during the division of blood cells^2^. While several genetic germline alleles and somatic alterations have been linked to the development of childhood ALL (e.g. *ETV6-RUNX1*, high hyperdiploidy), a large majority of cases do not have a dominant genetic component and may be attributable, at least in part, to environmental exposures. Several environmental exposures such as air pollution, radiation, and pesticide use have been previously associated with increased risk of childhood ALL^3^. Outdoor artificial light at night (ALAN), another environmental risk factor, has been linked to adult hematologic malignancies and circadian disruption^4–6^, but has yet to be extensively studied in childhood cancers.

ALAN contributes to circadian disruption by desynchronizing the sleep–wake cycle^7^. External light stimuli to the retina triggers a response in the suprachiasmatic nucleus in the hypothalamus. This response in turn modulates body temperature, cortisol, melatonin, and other hormones that are major pathways that bring about sleepiness. Light stimuli prior to sleep can delay the release of melatonin, increasing sleep latency and disrupting circadian rhythm^8^. The increase in exposure to outdoor ALAN over the past several years has been well documented and may be a contributing factor to poor sleep and overall health^9,10^. There is also evidence suggesting ALAN may play a stronger role in sleep disruption in children than in adults^11^.

Outdoor ALAN is, by nature, higher in urban environments, and therefore other exposures associated with urban environments need to be considered, a major one being air pollution. Childhood ALL risk has been associated with pollution from industrial sources^12^ and traffic, with a recent meta-analysis reporting a 9% increased risk for those residing in areas of higher traffic density^13^. Exposure to fine particulates, particulate matter less than 2.5 microns in aerodynamic diameter (PM_2.5_), has been linked to increased overall morbidity and mortality^14^, and a 2016 report by the World Health Organization International Agency for Research on Cancer classified the evidence for an association between PM_2.5_ and childhood ALL risk as suggestive^15^. To date, outdoor ALAN has not been examined in epidemiological studies of childhood ALL, and most existing studies on air population have small or moderate sample sizes^16,17^.

The aim of this study was to evaluate the association between ALAN, air pollution and risk of childhood ALL in a population-based case–control study in California.

## Methods

### Study population

The California Linkage Study of Early-Onset Cancers is a statewide linkage of birth records maintained by the California Department of Public Health and cancer diagnoses reported to the California Cancer Registry. The study was performed in accordance with relevant guidelines and regulations and the protocol was approved by the institutional review boards at the University of California, Berkeley, University of Southern California, and Yale University. This study was reviewed and approved by the Vital Statistics Advisory Committee and the institutional review board of the California Health and Human Services Agency and exempt from informed consent as data used was de-identified in accordance with California Code Sect. 102430 for the protection of human subjects that is approved by the federal Department of Health and Human Services and has a general assurance pursuant to Part 46 of Title 45 of the Code of Federal Regulations.

For the current analysis, eligible cases included all children who were born in California from 2000 through 2015 and were diagnosed with incident primary ALL between 2000 to 2015. ALL was defined as a diagnosis with an International Classification of Diseases for Oncology, 3rd edition, code of 9820, 9823, 9826, 9827, 9831–9837, 9940, or 9948. There were 2,819 cases initially identified, from which we excluded missing data on maternal residential address at the time of delivery (n = 6), maternal residence outside of California (n = 4), and missing data on congenital anomality or unknown information on congenital anomality (n = 27). For each case, we randomly selected 50 controls born in the same year from birth records. The final study population consisted of 2,782 childhood ALL cases and 139,100 controls.

### Exposure assessment

We assessed ALAN using the New World Atlas of Artificial Night Sky Brightness^18^, which provides a 750-m gridded spatial resolution global measure of luminance at the zenith (directly overhead) in millicandela per meter squared (mcd/m^2^). In contrast to using observations only captured by a satellite, which represents light that escapes upwards into the atmosphere, the World Atlas produces a better estimate of ground level light exposure because in includes measurements from handheld sky meters^19^. The New World Atlas was developed with observations from the National Oceanic and Atmospheric Administration Visible Infrared Imaging Radiometer Suite observations from May-December 2014. While this only provides a single 6-month average that was assigned retrospectively, the improved resolution of the sensor technology greatly reduce misclassification, and multiple studies have demonstrated stability in ALAN over time and high correlation between years in satellite estimates (R^2^ 0.92–0.98)^20–24^. ALAN was assigned to each case and control child by their geocoded maternal residential addresses at the time of delivery.

Air pollution was assessed with a validated United States (US) national model developed by Di et al^25^. Briefly, the model combines outputs from a chemical transport model with variables characterizing land use, population density, weather patterns, and satellite derived aerosol optical depth in an ensemble of machine learning models to estimate daily PM_2.5_ concentrations at a 1-km resolution. Validation of this model against air monitoring stations resulted in an R^2^ of 0.802 for the Pacific region. Cases were assigned the average PM_2.5_ concentration from birth until diagnosis. The exposure window for matched controls began at birth and ended when the corresponding case was diagnosed (e.g., if a case was diagnosed at 30 months, the matched controls were assigned the average PM_2.5_ from birth until 30 months of age).

### Other variables of interest

We abstracted characteristics from birth records, including birthweight, gestational age (22–36, 37–41, 42–44 weeks, or unknown), plurality (singleton or multiple), birth order (1st, 2nd, 3rd or higher), mode of delivery (cesarean section [C-section] or vaginal), complications during pregnancy, maternal history of miscarriage or stillbirth (yes/no), or history of previous cesarean section (no/yes/unknown). We also retrieved demographic and parental characteristics including sex, race/ethnicity, maternal age at delivery, mother’s place of birth, maternal education, and paternal age at delivery. We also linked maternal residential address to 2000 Census block group data to obtain the percentage of the population in the block group living below 150% of the federal poverty level. Of these factors, Hispanic ethnicity^1^, higher birthweight^26,27^, lower birth order^28,29^, caesarean delivery^30,31^, and older parental ages^32,33^, have been linked to an increased risk of childhood ALL, and the others are potential confounders for our analysis of ALAN and air pollution in relation to ALL risk.

### Statistical methods

**Figure 1:**
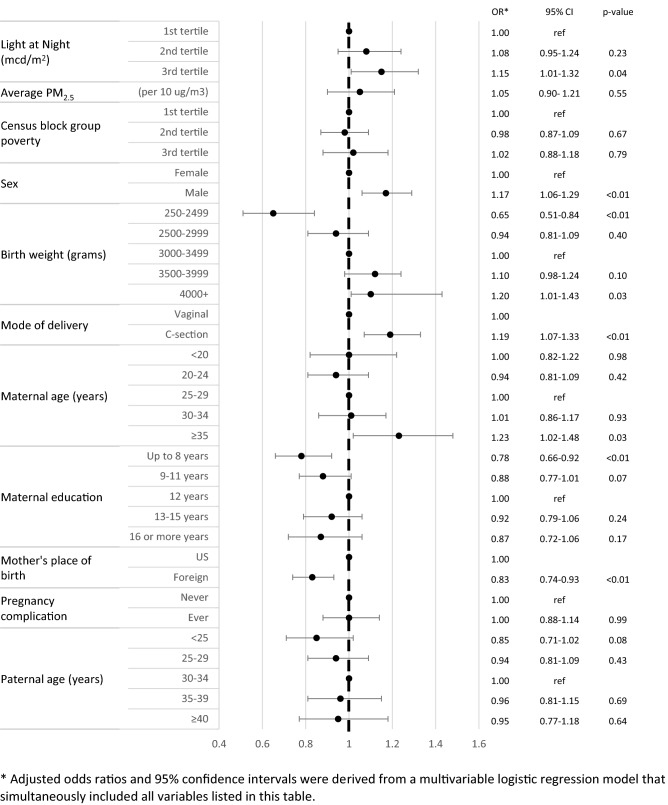
Association between outdoor artificial light at night, air pollution, birth characteristics, and risk of childhood acute lymphoblastic leukemia among Hispanic children in the California Linkage Study of Early-Onset Cancers. *Adjusted odds ratios and 95% confidence intervals were derived from a multivariable logistic regression model that simultaneously included all variables listed in this figure.

**Figure 2:**
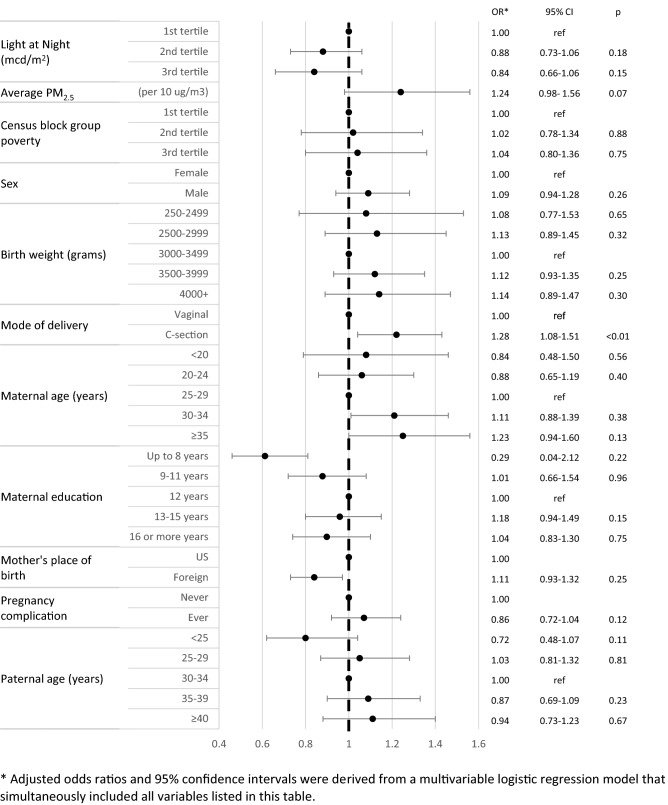
Association between outdoor artificial light at night, air pollution, birth characteristics, and risk of childhood acute lymphoblastic leukemia among Non-Hispanic White children in the California Linkage Study of Early-Onset Cancers. *Adjusted odds ratios and 95% confidence intervals were derived from a multivariable logistic regression model that simultaneously included all variables listed in this figure.

Spearman’s correlation was performed to evaluate correlation between ALAN and PM_2.5_ , with the former being the primary exposure of interest in this study and the latter being a potential confounder that has been linked to both ALAN and ALL risk in previous studies^13^. The associations between outdoor ALAN, PM_2.5_ and risk of childhood ALL were assessed using logistic regression models, and odds ratios (OR) and 95% confidence intervals (95% CI) were calculated. Examining the variables listed in Table 1, univariate ORs and their 95% CIs were obtained from bivariate logistic regression models with one independent variable at a time. A multivariable logistic regression model that included all variables was then fit, retaining those with *P* < 0.05 for the final model with the exception of outdoor ALAN and PM_2.5_, which were always included. The final model adjusted for percentage of population living below 150% of the poverty level in the census block group (tertiles), sex, race/ethnicity (non-Hispanic white, non-Hispanic black, Hispanic, Asian/Pacific Islander, other), birthweight (249–2499, 2500–2999, 3000–3499, 3500–3999, and ≥ 4000 g), mode of delivery (vaginal vs C-section), maternal age (< 20, 20–24, 25–29, 30–34, ≥ 35 years), maternal education (up to 8 years, 9–11 years, 13–15 years, ≥ 16 years, unknown), mother’s place of birth (US vs foreign countries), complications during pregnancy (yes/no), and paternal age (< 25, 25–29, 30–34, 35–39, ≥ 40 years, or unknown). Adjusted ORs and their 95% CIs were derived from the final model that simultaneously included these variables, outdoor ALAN, and PM_2.5_ (Tables 2, Figs. 1, 2). In sensitivity analyses, we further stratified cases based on their age of diagnosis (0–2, 3–7, 8–14 years).

**Table 1:** Distribution of outdoor artificial light at night, air pollution, and birth characteristics in the California Linkage Study of Early-Onset Cancers.

	Case		Control	
	n = 2782	%*	n = 139,100	%*
**Light at night (mcd/m**^**2**^**)**				
1st tertile	910	32.7	46,321	33.3
2nd tertile	900	32.4	46,478	33.4
3rd tertile	972	34.9	46,301	33.3
Median (IQR)	3.27 (1.83–5.92)		3.28 (1.85–5.70)	
**Average PM2.5 (µg/m**^**3**^**)**				
Mean (SD)	12.81 (3.88)		12.65 (3.88)	
**Census block group below 150% poverty**				
1st tertile	919	33.0	46,297	33.3
2nd tertile	904	32.5	46,435	33.4
3rd tertile	958	34.4	46,300	33.3
Unknown	1	0.04	68	0.05
**Sex**				
Female	1244	44.7	68,108	49.0
Male	1538	55.3	70,992	51.0
**Race/ethnicity**				
Non-Hispanic White	705	25.3	36,417	26.2
Non-Hispanic Black	89	3.2	7610	5.5
Hispanic	1656	59.5	76,196	54.8
Non-Hispanic Asian/Pacific Islander	280	10.1	16,643	12.0
Other	52	1.9	2234	1.6
**Birth weight (grams)**				
250–2499	140	5.0	9042	6.5
2500–2999	420	15.1	23,112	16.6
3000–3499	1063	38.2	54,466	39.2
3500–3999	855	30.7	39,866	28.7
4000 +	304	10.9	12,614	9.1
**Gestational age (weeks)**				
37–41	286	10.3	13,790	9.9
22–36	2200	79.1	110,499	79.4
42–44	153	5.5	7270	5.2
Unknown	143	5.1	7541	5.4
**Birth plurality**				
Singleton	2695	96.9	134,946	97.0
Multiple	87	3.1	4154	3.0
**Birth order**				
1st	1063	38.2	54,201	39.0
2nd	890	32.0	44,104	31.7
3rd and higher	829	29.8	40,795	29.3
**Mode of delivery**				
Vaginal	1867	67.1	98,178	70.6
C-section	915	32.9	40,922	29.4
**Year of birth**				
2000–2003	1025	36.8	51,250	36.8
2004–2007	892	32.1	44,600	32.1
2008–2015	865	31.1	43,250	31.1
**Maternal age (years)**				
< 20	234	8.4	12,966	9.3
20–24	563	20.2	31,316	22.5
25–29	737	26.5	36,553	26.3
30–34	713	25.6	34,376	24.7
≥ 35	535	19.2	23,889	17.2
**Maternal education**				
Up to 8 years	234	8.4	13,485	9.7
9–11 years	460	16.5	24,000	17.3
12 years	753	27.1	36,400	26.2
13–15 years	604	21.7	29,008	20.9
16 or more years	650	23.4	32,452	23.3
Unknown	81	2.9	3755	2.7
**Mother's place of birth**				
US	1290	46.4	62,090	44.6
Foreign	1492	53.6	77,010	55.4
**Miscarriage/stillbirth**				
Never	2269	81.6	114,918	82.6
Ever	513	18.4	24,139	17.4
Unknown	0		43	0.03
**Maternal complication during pregnancy**				
Never	2180	78.4	109,098	78.4
Ever	602	21.6	29,993	21.6
Unknown	0		9	0.01
**Previous C-section**				
Never	2390	85.9	120,489	86.6
Ever	392	14.1	18,611	13.4
**Paternal age (years)**				
< 25	455	16.4	26,200	18.8
25–29	620	22.3	30,717	22.1
30–34	727	26.1	33,702	24.2
35–39	491	17.6	23,278	16.7
≥ 40	313	11.3	15,356	11.0
Unknown	176	6.3	9847	7.1

* Percentages may not add up to 100 due to rounding.

**Table 2:** Association between outdoor artificial light at night, air pollution, birth characteristics, and risk of childhood acute lymphoblastic leukemia in the California Linkage Study of Early-Onset Cancers.

	Unadjusted*			Adjusted*		
	OR	95% CI	*p*-value	OR	95% CI	*p*-value
**Light at night (mcd/m**^**2**^**)**						
1st tertile	1.00			1.00		
2nd tertile	0.99	0.90–1.08	0.76	0.99	0.90–1.10	0.91
3rd tertile	1.07	0.98–1.17	0.15	1.05	0.95–1.17	0.33
**Average PM**_**2.5**_						
(Per 10 µg/m^3^)	**1.11**	**1.01–1.22**	**0.04**	1.08	0.96–1.20	0.20
**Census block group below 150% poverty**						
1st tertile	1.00			1.00		
2nd tertile	0.98	0.89–1.08	0.68	0.97	0.88–1.07	0.58
3rd tertile	1.04	0.95–1.14	0.37	1.03	0.93–1.15	0.57
Unknown	0.74	0.10–5.34	0.77	0.73	0.10–5.30	0.76
**Sex**						
Female	1.00			1.00		
Male	**1.19**	**1.10–1.28**	** < 0.01**	**1.17**	**1.08–1.26**	** < 0.01**
**Race/ethnicity**						
Non-hispanic White	1.00			1.00		
Black	**0.60**	**0.48–0.75**	** < 0.01**	**0.65**	**0.52–0.82**	** < 0.01**
Hispanic	**1.12**	**1.03–1.23**	**0.01**	**1.29**	**1.16–1.43**	** < 0.01**
Asian	**0.87**	**0.76–1.00**	**0.05**	0.93	0.80–1.08	0.34
Other	1.20	0.90–1.60	0.20	1.26	0.90–1.77	0.17
**Birth weight (grams)**						
250–2499	**0.79**	**0.66–0.95**	**0.01**	**0.77**	**0.64–0.92**	** < 0.01**
2500–2999	0.93	0.83–1.04	0.22	0.95	0.85–1.06	0.37
3000–3499	1.00			1.00		
3500–3999	**1.10**	**1.00–1.20**	**0.04**	1.07	0.97–1.17	0.17
4000 +	**1.24**	**1.09–1.40**	** < 0.01**	**1.15**	**1.01–1.31**	**0.03**
**Mode of delivery**						
Vaginal	1.00			1.00		
C-section	**1.18**	**1.09–1.27**	** < 0.01**	**1.16**	**1.07–1.26**	** < 0.01**
**Maternal age (years)**						
< 20	0.90	0.77–1.04	0.14	0.99	0.83–1.19	0.95
20–24	**0.89**	**0.80–1.00**	**0.04**	0.93	0.83–1.06	0.27
25–29	1.00			1.00		
30–34	1.03	0.93–1.14	0.59	1.04	0.93–1.16	0.51
≥ 35	1.11	0.99–1.24	0.07	**1.16**	**1.01–1.32**	**0.04**
**Maternal education**						
Up to 8 years	**0.84**	**0.72–0.97**	**0.02**	**0.76**	**0.65–0.89**	** < 0.01**
9–11 years	0.93	0.82–1.04	0.20	0.90	0.79–1.01	0.08
12 years	1.00			1.00		
13–15 years	1.01	0.90–1.12	0.91	1.01	0.90–1.13	0.87
16 or more years	0.97	0.87–1.08	0.55	0.96	0.85–1.09	0.52
Unknown	1.04	0.83–1.31	0.72	0.99	0.75–1.30	0.93
**Mother's place of birth**						
US	1.00	US		1.00		
Foreign	0.93	0.86–1.01	0.07	**0.90**	**0.82–0.98**	**0.02**
**Maternal complication during pregnancy**						
Never	1.00			1.00		
Ever	1.00	0.92–1.10	0.92	1.03	0.93–1.13	0.60
**Paternal age (years)**						
< 25	**0.81**	**0.72–0.91**	** < 0.01**	**0.82**	**0.71–0.95**	** < 0.01**
25–29	0.94	0.84–1.04	0.23	0.94	0.84–1.06	0.30
30–34	1.00			1.00		
35–39	0.98	0.87–1.10	0.70	0.95	0.84–1.07	0.43
≥ 40	0.94	0.83–1.08	0.41	0.91	0.79–1.06	0.23
Unknown	**0.83**	**0.70–0.98**	**0.03**	0.88	0.74–1.06	0.17

*Unadjusted odds ratios and 95% confident intervals were obtained from bivariate logistic regression models that included one independent variable at a time. Adjusted odds ratios and 95% confidence intervals were derived from a multivariable logistic regression model that simultaneously included all variables listed in this table.

Significant values are in [bold].

All analyses were performed using SAS (version 9.4, SAS Institute, Inc., Cary, North Carolina) and all tests were two-sided with an alpha value of 0.05.

## Results

The median ALAN level was 3.27 mcd/m^2^ (inter-quartile range, IQR 1.83–5.92) in cases and 3.28 mcd/m^2^ (IQR 1.85–5.70) in controls (Table 1). The mean PM_2.5_ level was 12.81 µg/m^3^ (standard deviation, SD 3.88) in cases and 12.65 µg/m^3^ (SD 3.88) in controls. ALAN and air pollution were moderately correlated with a Spearman coefficient of 0.52. There were more male cases (55.3%) than females. A majority of the study population was Hispanic (59.5% cases and 54.8% controls). ALAN and PM_2.5_ were significantly higher in non-Hispanic White children (median ALAN 2.38 mcd/m^2^, mean PM_2.5_ 11.47 µg/m^3^) than in Hispanic children (median ALAN 2.03 mcd/m^2^, mean PM_2.5_ 13.34 µg/m^3^, Supplemental Table 1).

Among the overall study population, we did not observe a clear association between outdoor ALAN and childhood ALL (OR_3rd tertile_ 1.07, 95% CI 0.98–1.17) (Table 2). We did observe an association between PM_2.5_ and ALL risk (OR 1.11, 95% CI 1.01–1.22) that was no longer significant after adjusting for other covariates (adjusted OR 1.08, 95% CI 0.96–1.20). There was increased risk of childhood ALL in male children compared to female (adjusted OR 1.17, 95% CI 1.08–1.26). Compared to non-Hispanic White children, Hispanic ethnicity was associated with increased ALL risk (adjusted OR 1.29, 95% CI 1.16–1.43) while non-Hispanic Black (adjusted OR 0.65, 95% CI 0.52–0.82) children had a lower risk. Compared to children born at a normal birthweight (3000–3499 g), low birthweight (< 2500 g) was associated with decreased ALL risk (adjusted OR_<2500 g_ 0.77, 95% CI 0.64–0.92) while high birthweight (> 4000 g) was associated with increased ALL risk (adjusted OR_>4000 g_ 1.15, 95% CI 1.01–1.31). Delivery by C-section was associated with a 1.16 (95% CI 1.07–1.26) increased risk of ALL. Mothers older than 35 years were more likely to have children who developed ALL compared to mothers between 25–29 years (adjusted OR 1.16, 95% CI 1.01–1.32). Children with fathers under age 25 years were less likely to have ALL compared to those with fathers between the age of 30–34 years (adjusted OR 0.82, 95% CI 0.71–0.95). Compared to mothers who completed high school, those with less than 8 years of school were less likely to have children who developed ALL (adjusted OR 0.76, 95% CI 0.65–0.89). Children of foreign-born mothers were less likely to develop ALL compared to US born mothers (adjusted OR 0.90, 95% CI 0.82–0.98).

Among Hispanic children, there was a 15% increased risk of childhood ALL (95% CI 1.01–1.32) for those residing in the highest tertile of ALAN compared to the lowest (Fig. 1). In non-Hispanic White children, there did not appear to be an association between exposure to outdoor ALAN and ALL risk (adjusted OR_3rd tertile_ 0.84, 95% CI 0.66–1.06, Fig. 2). The association between PM_2.5_ and ALL appeared to be stronger in non-Hispanic White children (adjusted OR 1.24, 95% CI 0.98–1.56) than Hispanic children (adjusted OR 1.05, 95% CI 0.90–1.21). Delivery by C-section was the only covariate still significantly associated with increased ALL risk in non-Hispanic White children (adjusted OR 1.28, 95% CI 1.08–1.51).

When stratified by age, the risk association of ALAN and ALL was highest for the oldest age group with evidence of an increasing trend from 1.00 (95% CI 0.84–1.19, Supplemental Table 2 and Supplemental Fig. 1) in the 0–2 years age group to 1.22 (95% CI 0.88–1.69) in the 8–14 years age group. Foreign maternal place of birth was only protective in the 0–2 years age group (adjusted OR_0-2 years_ 0.84, 95% CI 0.73–0.97). Compared to non-Hispanic White children, the risk among non-Hispanic Black children was lower in the 0–2 years age group (adjusted OR_0-2 years_ 0.60, 95% CI 0.41–0.87) and 3–7 years age group (adjusted OR_3-7 years_ 0.61, 95% CI 0.44–0.85) and null in the oldest age group. In contrast, the risk in Hispanic children was not significant in the 0–2 years age group (adjusted OR_0-2 years_ 1.17, 95% CI 0.98–1.38) but increased in successive older age groups (adjusted OR_3-7 years_ 1.33, 95% CI 1.14–1.54, adjusted OR_8-14 years_ 1.60, 95% CI 1.16–2.22). Birthweight was also only significantly associated with ALL risk in the 0–2 years age group (adjusted OR_<2500 g_ 0.52, 95% CI 0.37–0.74).

## Discussion

In this large, population-based case–control study, exposure to outdoor ALAN was associated with increased childhood ALL risk, but only among Hispanic children. These results are similar to those reported in adult leukemia^5^. Compared to many ecological studies on the association between ALAN and cancer risk, we were able to adjust for important confounders, including birth characteristics, parental factors, markers of socioeconomic status, and air pollution. Since air pollution is highly correlated with ALAN and has been previously linked to the etiology of childhood ALL^13^, we accounted for it as a potential confounder. The magnitude of association between PM_2.5_ and ALL risk in our study (OR 1.08; 95% CI 0.96–1.20) was consistent with what was reported in a meta-analysis by Filippini et al. (OR 1.11; 95% CI 0.95–1.31)^13^. PM_2.5_ did not appear to be a confounder of ALAN in multivariable analyses, suggesting that the association between ALAN exposure and childhood ALL risk was independent of air pollution.

Exposure to light prior to sleep leads to suppression of melatonin and delayed onset of sleep^34–37^. Melatonin is involved in the nuclear transcription factor kappa beta pathway^38^, which modulates levels of many immune cytokines, such as interleukin-8 and interleukin-10, that have been previously implicated in the etiology of childhood ALL^39,40^. Disruptions to sleep can also alter inflammatory response from Toll-like receptors^41^, which are involved in hematopoiesis and leukeogenesis^42^. We observed the strongest association between ALAN and ALL in the older 8–14 years age group (Supplemental Fig. 1), so it may be the continued disruption to these pathways over time that contribute to development of leukemia. Additional studies have also linked ALAN exposure to circadian disruption^43^ and tumor progression through potential epigenetic pathways^44,45^. Several genes involved in circadian rhythm have been found to be differentially expressed in ALL patients as well^46^. As our study is the first to report a novel association between exposure to outdoor ALAN and childhood ALL risk, it would be helpful to further examine the role of ALAN in other epidemiological studies of childhood ALL and conduct additional mechanistic evaluations.

Prior studies on ALL have demonstrated differential risk patterns between Hispanic and non-Hispanic White children – including risk factors such as daycare attendance, infections, diet, and genetics^47–50^. Hispanic children have the highest risk of ALL among all racial/ethnic groups in California and the US^51^, supporting a possibly different etiological profile compared to non-Hispanic Whites. For instance, compared to non-Hispanic White children in our study, Hispanic children resided in areas of greater PM_2.5_ and ALAN (Supplemental Table 1). Differences in genetics may also explain how the environment influences the way certain individuals experience ALAN and disruptions in circadian rhythm. Studies have reported shorter sleep durations in minority populations compared to non-Hispanic Whites^52^. It could be a combination of both environment and genetics as interactions have been observed for air pollution and other conditions such as cardiovascular disease^53^ and Parkinson’s disease^54^. There may also be interactions with other attributes of the built environment that we did not evaluate, such as green space, which has been shown to improve health^55^ and attenuate the risks observed with air pollution exposure^56,57^, especially in communities of lower socioeconomic status^58^.

Strengths of our study include the large, population-based sample that we were able to assemble by linking statewide cancer diagnosis information to the statewide birth records in California, the most populous state in the US. The California Cancer Registry is a part of the Surveillance, Epidemiology, and End Results program and provides high quality and comprehensive ascertainment of cancer cases diagnosed in the state. Linkage to the birth records provides information on many important covariates encompassing known/suspected risk factors for childhood ALL and other potential confounders for our analysis of ALAN and air pollution. Notably, no cases or controls had to be contacted for participation in the study, minimizing selection bias. In addition, we derived high resolution estimates of ALAN and PM_2.5_ based on maternal residential address at the time of delivery, which was documented in birth records prior to the diagnosis of childhood ALL, therefore reducing recall bias.

A primary limitation of this study is the lack of data on residential history. While we do have additional address information for cases at time of ALL diagnosis, the corresponding information for the reference date (age at which the matched case was diagnosed) is not available for our controls. Residential mobility appears to be greater in early childhood, though most moves tend to be to similar neighborhoods^59,60^. In addition, we have no reason to believe that residential mobility, in regards to ALAN or PM_2.5_ exposure, would differ systematically between cases and controls. Another limitation is that the measure of outdoor ALAN that we used was based on observations from 2014 and consisted of an estimate of luminance at the zenith. It did not characterize light at various wavelengths, which may be an important distinction. Animal studies show that shorter, blue spectrum wavelength light (450–500 nm) was more detrimental to sleep^8,61^, which could be an issue as cities are moving away from amber lights towards more shorter wavelength LED (light emitting diode) streetlights^44^. While our use of the New World Atlas provides a better estimate of ALAN exposure at a more precise 750-m grid (compared to the 2.7-km Defense Meteorological Satellite Program Operational Linescan System) , McIsaac et al. recently demonstrated the misclassification that is still present in such large grids and how recent advances may further improve the accuracy of exposure estimate^20^. Another important constraint is that we do not have any data on indoor exposure to light at night, which could be influenced by curtains or other light blocking accessories, type of windows and glasses, and type of indoor light sources. A comparison of outdoor ALAN and personal exposure in children in the Netherlands only found a weak correlation of 0.31^62^. However, disruptions to levels of melatonin have been shown to persist hours after exposure to light stimuli^34,35^. The primary exposure of interest in this study is outdoor ALAN, not indoor ALAN or all ALAN. Hopefully, our work can inspire investigators of future studies to expand the scope of exposure assessment to include all types of ALAN.

With regard to air pollution, we recognize that a limitation of our study is the inability to evaluate the composition of particulate matter, e.g., air pollution from farming may contain pesticides as opposed to carbon and brake dust from road traffic. Elucidation of the components of air pollution is needed to better understand the constituents behind the risk association between air pollution and childhood ALL. A recent review found levels of outdoor PM_2.5_ to be correlated with indoor PM_2.5_, though it varied depending upon the composition^63^. Lastly, despite our ability to adjust for many known/suspected risk factors of childhood ALL, residual confounding due to imprecise measurements or unmeasured factors (e.g., exposure to smoking) remains a possibility.

In summary, exposure to outdoor ALAN was associated with an increased risk of ALL among Hispanic children. Future studies of ALAN and childhood cancers will require additional assessment of indoor ALAN and the relationship between ALAN and sleep. Inclusion of genetics may identify potential gene-environment interactions that cause individuals to differentially respond to their environment. This is especially important in Hispanic children, a subgroup of the population in which childhood ALL rates have been increasing in recent years^1^.

## Supplementary Information

Supplementary Information.

## Data Availability

The datasets generated during and/or analyzed during the current study are not publicly available due to data sharing policies set by the state of California. We welcome questions from other investigators or requests for additional analyses that are pertinent to the data presented in this manuscript, and potential data sharing when permitted by the California Health and Human Services Agency Committee for the Protection of Human Subjects.
