# Supplementary material for: Outdoor artificial light at night, air pollution, and risk of childhood acute lymphoblastic leukemia in the California Linkage Study of Early-Onset Cancers
Source: Sci Rep. 2023 Jan 11;13:583. doi: 10.1038/s41598-022-23682-z (PMC9834257; doi:10.1038/s41598-022-23682-z)
Supplement: Supplementary file 1 — Supplementary Information. [file 41598_2022_23682_MOESM1_ESM.pdf]

Supplemental Table 1. Distribution of light at night and air pollution by race/ethnicity in controls in the California Linkage Study of Early-Onset Cancers

		Non-Hispanic White<br>n = 36,417	Hispanic<br>n = 76,196	p-value
**Light at Night**	**Median (IQR)**	2.38 (1.26-3.83)	3.80 (2.03-6.66)	<0.01
**Average PM2.5**	**Mean (SD)**	11.47 (3.77)	13.34 (3.90)	<0.01

Supplemental Table 2. Multivariate association between light at night, air pollution, and birth outcomes with acute lymphoblastic leukemia risk in the California Linkage Study of Early-Onset Cancers by age

		Age 0-2 (N cases = 1025, N controls = 51,250)			Age 3-7 (N cases = 1423, N controls = 71,150)			Age 8-14 (N cases = 334, N controls = 16,700)		
		OR	95% CI	p	OR	95% CI	p	OR	95% CI	p
**Light at Night**										
	**1st tertile**	1.00			1.00			1.00		
	**2nd tertile**	1.00	0.86- 1.17	0.96	0.97	0.85- 1.11	0.67	1.07	0.80- 1.43	0.66
	**3rd tertile**	1.00	0.84- 1.19	0.99	1.06	0.91- 1.23	0.46	1.22	0.88- 1.69	0.24
**PM2.5**										
	**Continous (per 10 ug/m^3)**	1.09	0.93- 1.29	0.30	1.09	0.93- 1.29	0.29	0.89	0.59- 1.36	0.60
**Census block group below 150% poverty**	**1st tertile**	1.00			1.00			1.00		
	**2nd tertile**	0.88	0.75- 1.03	0.10	1.03	0.90- 1.18	0.67	1.03	0.78- 1.35	0.84
	**3rd tertile**	0.94	0.79- 1.12	0.48	1.10	0.95- 1.28	0.22	1.02	0.74- 1.41	0.89
	**Unknown**				1.89	0.26- 13.99	0.53			
**Sex**	**Female**	1.00			1.00			1.00		
	**Male**	1.19	1.05- 1.35	<.01	1.11	1.00- 1.23	0.06	1.35	1.08- 1.69	<.01
**Race/ethnicity**	**White**	1.00			1.00			1.00		
	**Black**	0.60	0.41- 0.87	<.01	0.61	0.44- 0.85	<.01	1.03	0.57- 1.84	0.93
	**Hispanic**	1.17	0.98- 1.38	0.08	1.33	1.14- 1.54	<.01	1.60	1.16- 2.22	<.01
	**Asian**	0.92	0.72- 1.16	0.47	0.93	0.75- 1.15	0.49	0.95	0.58- 1.55	0.83
	**Other**	1.27	0.74- 2.17	0.39	1.16	0.73- 1.85	0.53	1.97	0.64- 6.09	0.24
**Birth weight (grams)**	**250-2499**	0.52	0.37- 0.74	<.01	0.84	0.65- 1.07	0.15	1.18	0.77- 1.83	0.44
	**2500-2999**	0.87	0.72- 1.06	0.16	1.02	0.87- 1.19	0.79	0.86	0.61- 1.22	0.40
	**3000-3499**	1.00			1.00			1.00		
	**3500-3999**	1.13	0.97- 1.31	0.12	1.04	0.92- 1.19	0.50	0.97	0.74- 1.27	0.81
	**4000+**	1.19	0.96- 1.47	0.10	1.08	0.90- 1.31	0.41	1.23	0.86- 1.75	0.25
**Mode of delivery**	**Vaginal**	1.00			1.00			1.00		
	**C-section**	1.22	1.04- 1.43	0.01	1.15	1.00- 1.31	0.05	1.57	1.21- 2.05	<.01
**Maternal age (years)**	**<20**	1.08	0.79- 1.46	0.64	0.97	0.76- 1.24	0.81	0.79	0.49- 1.27	0.33
	**20-24**	1.06	0.86- 1.30	0.58	0.85	0.72- 1.01	0.07	0.91	0.65- 1.26	0.55
	**25-29**	1.00			1.00			1.00		
	**30-34**	1.21	1.01- 1.46	0.04	1.01	0.86- 1.18	0.93	0.78	0.56- 1.07	0.12
	**>=35**	1.25	1.00- 1.56	0.05	1.25	1.03- 1.50	0.02	0.71	0.47- 1.07	0.11
**Maternal education**	**Up to 8 years**	0.61	0.46- 0.81	<.01	0.78	0.63- 0.97	0.03	1.09	0.72- 1.64	0.69
	**9-11 years**	0.88	0.72- 1.08	0.21	0.83	0.70- 0.98	0.03	1.29	0.92- 1.79	0.13
	**12th years**	1.00			1.00			1.00		
	**13-15 years**	0.96	0.80- 1.15	0.64	1.01	0.87- 1.18	0.87	1.16	0.83- 1.61	0.39
	**16 or more years**	0.90	0.74- 1.10	0.29	0.97	0.82- 1.15	0.73	1.06	0.73- 1.54	0.74
	**Unknown**	0.90	0.57- 1.40	0.63	1.15	0.80- 1.65	0.45	0.37	0.10- 1.32	0.12
**Mother's place of birth**	**US**	1.00			1.00			1.00		
	**Foreign**	0.84	0.73- 0.97	0.01	0.95	0.84- 1.07	0.38	0.86	0.66- 1.14	0.30
**Maternal complication during pregnancy**	**Never**	1.00			1.00			1.00		
	**Ever**	1.07	0.92- 1.24	0.37	1.01	0.89- 1.15	0.88	0.94	0.69- 1.26	0.67
**Paternal age (years)**	**<25**	0.80	0.62- 1.04	0.09	0.86	0.70- 1.06	0.16	0.70	0.47- 1.05	0.08
	**25-29**	1.05	0.87- 1.28	0.60	0.93	0.79- 1.09	0.36	0.71	0.51- 0.99	0.04
	**30-34**	1.00			1.00			1.00		
	**35-39**	1.09	0.90- 1.33	0.39	0.85	0.72- 1.01	0.07	1.00	0.70- 1.42	0.99
	**>=40**	1.11	0.88- 1.40	0.39	0.80	0.65- 0.98	0.03	0.85	0.54- 1.34	0.48
	**Unknown**	0.82	0.60- 1.11	0.20	0.90	0.70- 1.16	0.42	0.95	0.60- 1.52	0.83

Supplemental Figure 1. Multivariate association between light at night, air pollution, and birth outcomes with acute lymphoblastic leukemia risk in the California Linkage Study of Early-Onset Cancers by age

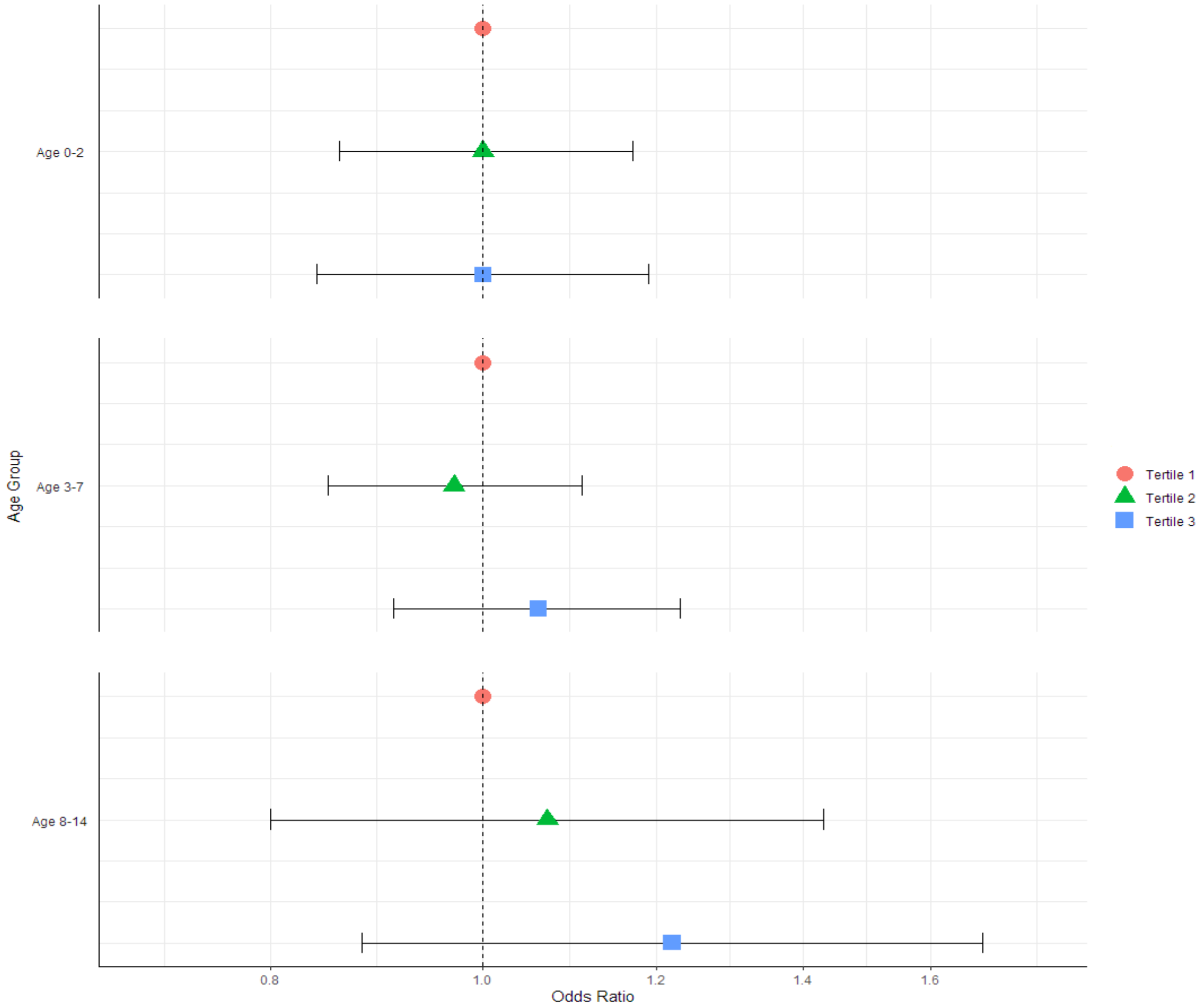

Adjusted for Census block group below 150% poverty, sex, race/ethnicity, birthweight, mode of delivery, maternal age, maternal education, mother's place of birth, history of complications during pregnancy, and paternal age
